# The “Free by 5” Campaign for Universal, Free Antiretroviral Therapy

**DOI:** 10.1371/journal.pmed.0020227

**Published:** 2005-07-19

**Authors:** Alan Whiteside, Sabrina Lee

## Abstract

The Free by 5 declaration, launched in November 2004, aims to achieve free access for all individuals with HIV to a comprehensive medical package including antiretroviral treatment.

The Free by 5 declaration (http://www.ukzn.ac.za/heard/freeby5/freeby5.htm), launched in November 2004, is a campaign to achieve free access for all individuals with HIV to a comprehensive minimum medical package including antiretroviral treatment (ART). The declaration was developed in response to the World Health Organization (WHO) and the Joint United Nations Programme on HIV/AIDS (UNAIDS) “3 by 5” strategy, which aims to scale up access to ART to ensure that 3 million people have access to ART by the end of 2005 [1]. We believe that the declaration made an important contribution to the debate on provision of ART in resource-poor settings.

A number of debates still surround the 3 by 5 strategy, including how it will be operationalised and funded [1]. Questions remain over how the poorest and most vulnerable groups can be reached and how a suitable level of adherence to the drugs can be achieved to avoid drug resistance [2].

## The Problem with User Fees

Currently, fewer than 8% of people living with HIV/AIDS in Africa are on ART [3]. While user fees represent only one of the barriers to access to essential ART, the Free by 5 initiative firmly states that unless treatment is provided free of charge to *all* in developing countries, 3 by 5 will struggle to meet its ambitious target. Many countries still impose user fees for ART and associated tests, and as AIDS is an impoverishing disease, this means treatment is unaffordable for most [4].

We must state clearly that we acknowledge that providing treatment free of charge is not the only precondition for the scale-up of treatment programmes. In most developing countries, the availability and efficiency of health infrastructure is the dominant obstacle to effective health care. Adequate primary health-care infrastructure and staff is fundamental to the provision of treatment programmes. However, we argue that where drugs and services are administered, providing treatment for free would assist patients to gain greater access to, remain adherent to, and avoid instability in treatment regimens. Simply stated, the Free by 5 campaign maintains that user fees are an additional and unnecessary obstacle to treatment access and the efficiency and equity of treatment programmes in the context of this major health crisis. Furthermore, removal of patient fees as a significant barrier to access is realistic and feasible in resource-poor settings [Fig pmed-0020227-g001]

**Figure pmed-0020227-g001:**
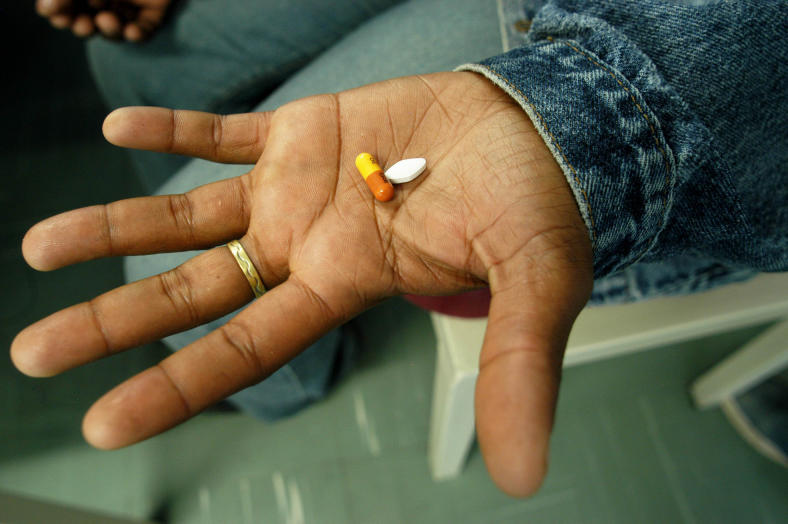
Antiretroviral drugs are unaffordable to most patients in Africa (Photo: Copyright WHO/Eric Miller)]

This article outlines the Free by 5 campaign and its objectives. It sketches the arguments for free treatment and describes how the initiative influenced the debate surrounding HIV/AIDS treatment among donors, international institutions, non-government organisations, professionals in the fields of public health and economics, and the concerned public.

## Origins and Objectives of Free by 5

The Free by 5 initiative began as a challenge to one of us (Alan Whiteside) at a Commission on HIV/AIDS and Governance in Africa meeting in Maputo from Veronique Collard, an academic and activist, who asked, “What do economists believe?” This challenge led to the idea that a declaration, giving the economists' position, could be adopted by the International AIDS Economics Network meeting in Bangkok. A first draft was prepared and presented at this meeting, but the reception from the assembled economists was underwhelming.

Despite this, we decided to press ahead. Veronique gave freely of her time in developing the initiative and was guided by many people, particularly Bernard Taverne of Institut de Recherche pour le Développement, Senegal; Alice Desclaux of University of Paul Cezanne, France; Gorik Ooms of Médecins Sans Frontières (MSF) in Belgium; and Alan Whiteside. We at HEARD contributed to the costs involved and provided a secretariat. Countless others in the field gave their support and enthusiasm, without which the initiative could not have happened.

The overall goal of the initiative was to gather support from professionals and organisations to promote universal free access to a minimum health-care package, including ART, for people with HIV. It aimed to lobby international institutions, including UNAIDS, WHO, and donors— such as the Global Fund, the World Bank, and the US President's Emergency Plan for AIDS Relief—to adopt guidelines and actively promote the principle and implementation of free treatment. Donors were urged to pledge additional resources to ART through long-term commitments. Finally, the initiative sought to provide economic and public health evidence to inform the decisions of policy makers and governments and assist activists in their advocacy efforts.

## What Do WHO and 3 by 5 Say about Free Treatment?

The Free by 5 declaration makes the point that the 3 by 5 strategy is unrealistic. Although the WHO seeks increased access to ART for all people living with HIV/AIDS, it does not address the costs at the patient level. The Free by 5 campaign believes that free treatment is an absolute prerequisite to the scale-up of treatment programmes and universal access to treatment.

User fees inhibit patient adherence.

Despite clear indications that patient fees inhibit access to treatment programmes, as outlined in the following section, the WHO strategy documents do not address this issue. Instead, the WHO treatment guidelines make frequent references to “affordability.” The WHO strategy published in 2003 recommended making antiretrovirals affordable and providing them free of charge to the poor [5]. A revision of the guidelines recommends providing “medication free of charge to those who can least afford treatment through subsidized or other financing strategies” [6]. A 2004 Consultation Report stated that if “cost recovery schemes prove inefficient or obstructive to access, free delivery to all should be considered” [7]. It should be noted that much of this debate is confined to ART. In the Free by 5 declaration, we recognise that treatment includes testing, laboratory examinations, and associated drugs.

## Arguments for and against Free Treatment

What affordability means and who is poor is not defined in these WHO guidelines. Defining the poor by income level is problematic in countries where the informal economy (that is, unregistered, unrecognised, and unsupported employment) dominates and income records are poor.

The process of implementing exemptions based on income is a waste of scarce financial and human resources as systems are costly to put in place and administer. Exemptions or waivers rarely reach those who are eligible to receive them [8]. While the WHO maintains that “free treatment would be difficult to implement in many health systems” [9], the Free by 5 declaration states that it will be easier and more cost-effective to provide treatment to all patients free of charge.

Countries set their own criteria for access, and these vary. In addition, perceptions of equity vary among and between governments, donors, and activists. These variations are difficult to manage from a clinical perspective and prevent equity from being attained at a national and international level [10]. Existing criteria for access are inequitable: a first-come, first-served basis favours the rich, more educated, and urban people. Universal free treatment is necessary to achieve equity in access and to avoid exclusion of the most susceptible and vulnerable groups.

The Free by 5 declaration details a number of arguments that have been made against free treatment and gives evidence that counter these views.

There are claims that patients should pay in order to give value to treatment and remain adherent to the drugs. Studies in Senegal have shown that user fees inhibit patient adherence and cause frequent interruptions in therapy [11], and in Kenya user fees have led to the discontinuation of treatment and delays in health-seeking behaviour [12]. The negative relationship between end-user costs and adherence has also been echoed in data from Uganda [13], Nigeria [14], Botswana [15], and the Côte d'Ivoire [16]. When ART must be paid for, patients are also more likely to misuse drugs and purchase them on the informal market. This ultimately leads to drug resistance. In some cases, the costs of laboratory tests deter people from joining treatment programmes. Providing treatment for free reduces delays in seeking care and improves adherence and may influence the quality of care.

A major argument against free treatment is that of AIDS exceptionalism. In other words, why should AIDS be treated for free when others diseases are not? There are three simple arguments countering this view.

First, AIDS is the worst epidemic humanity has ever faced, which has devastating long-term social, economic, and personal impacts and is a major obstacle to development. The exceptionalism of the disease requires exceptionalism in the response. At the UN General Assembly meeting on HIV/AIDS in New York, on 22 September 2003, WHO Director General Jong-Wook Lee described the lack of ART as a global health emergency. Second, other diseases are treated free where there is a public health reason to do so. Third, it is feasible to implement free HIV/AIDS treatment in resource-poor settings. Given the nature of the AIDS epidemic, providing free treatment should be an imperative even if it can not be applied to all diseases or all in need.

It is argued that patient fees are necessary to ensure the sustainability of treatment programmes. However, in Senegal fees amount to little more than 10% of the cost of drugs [11]. Patient contributions do not cover other costs such as staff, training, and social services. Sustainability can be achieved only through long-term commitments from donors and governments. The WHO and UNAIDS estimate that the total cost of providing treatment through the 3 by 5 initiative for 2005 is $3.8 billion, and this will increase to $6.7 billion in 2007 [17]. The contribution made by fee-paying patients is negligible. In Ghana, for instance, user fees amount to no more than a tiny fraction of the Ministry of Health aggregate budget [18]. Therefore, providing treatment for free will have virtually no effect on global resource needs. Significant resources still need to be mobilised.

AIDS is the worst epidemic humanity has ever faced.

## The Impact of Free by 5

The Free by 5 declaration, which is available in French, English, and Spanish, was disseminated worldwide through global MSF offices, universities, schools of public health, and NGO networks. It was signed by more than 600 people, many of whom are respected public health professionals, economists, policy makers, and key activists. Some of the influential signatories are shown in Box 1. The declaration was also signed by a number of organisations, shown in Box 2.

**Box 1. Some of the Influential Signatories of Free by 5**

Stephen Lewis, UN Special Envoy on HIV/AIDS

Helene Rossier-Blavier, Director General of AIDES France and Vice-President of the Global Fund

Hoosen Coovadia, Victor Daitz Chair in HIV/AIDS Research, University of KwaZulu-Natal

Philippe Douste Blazy, French Minister of Health

Bernard Kouchner, Founder of Médecins Sans Frontières, former French Health Minister

Omar Kabbaj, President of the African Development Bank, Tunis

Max Essex, Chair, Harvard School Public Health AIDS Initiative

**Box 2. Some of the Organizations That Signed the Free by 5 Declaration**

Norwegian Council for Africa

International HIV/AIDS Alliance

Treatment Action Campaign

National Agency for AIDS Research (ANRS) France

Réseau Accès aux Médicaments Essentiels (RAME), Burkina Faso

Fondation Femme Plus, DRC

The initiative sparked extensive debate among Internet-based development and public health fora. It culminated in a media release, which was disseminated widely among the global press. The initiative was picked up by British, French, South African, and Kenyan national newspapers as well as the UN IRIN Plus News. It was also featured on a number of Web sites.

It is difficult to asses the impact of the declaration over such a short time frame. After the media launch, the declaration and list of signatures were sent to UNAIDS, WHO, and the World Bank. We urged these organisations to give unambiguous support to the implementation of free treatment and take a lead in raising awareness about the issue. We encouraged all governmental and nongovernmental actors to adopt universal free treatment and actively promote its implementation. We asked that the issue be included on the agenda of technical meetings and political forums planned in the framework of the 3 by 5 initiative, and reflected clearly in all WHO/UNAIDS guidelines.

At the very least, the Free by 5 declaration publicised the issue and the importance of universal free access to treatment and created a debate surrounding free treatment in public health, health economics, and human rights circles throughout the world. It is an issue that has been placed on the international agenda for discussion.

For instance, the Free by 5 campaign played a role in inspiring the recent WHO/UNAIDS/World Bank meeting entitled “Ensuring universal access: User fees and free care polices in the context of HIV treatment” (21–23 March 2005). The meeting acknowledged that “for many individuals in poor countries, affordability poses an insurmountable obstacle” that depresses uptake of AIDS treatment programmes and decreases adherence of those enrolled. The meeting also noted that user fees contribute very little to overall sustainability and that if AIDS care and treatment programmes are to be scaled up, a broad shift to other financing models is required [19].

## Conclusion

There are many lessons from this initiative, but two are particularly important. The first is that a good idea can, with the right support, be turned into something concrete. The second is that goodwill and good sense are as important as money in shaping the policy environment. It was a small initiative with a big impact. We hope that eventually we will see the fruits of our efforts: universal free access to ART to all who need them. We feel privileged to have been part of this important campaign.

## Abbreviations

ART: antiretroviral treatment
UNAIDS: Joint United Nations Programme on HIV/AIDS
WHO: World Health Organization
